# Infrared Laser Ablation Microsampling with a Reflective Objective

**DOI:** 10.1021/jasms.1c00306

**Published:** 2022-02-01

**Authors:** Chao Dong, Luke T. Richardson, Touradj Solouki, Kermit K. Murray

**Affiliations:** † Department of Chemistry, Louisiana State University, Baton Rouge, Louisiana 70803, United States; ‡ Department of Chemistry and Biochemistry, Baylor University, Waco, Texas 76706, United States

**Keywords:** infrared laser, laser ablation, reflective objective, microsampling, proteomics

## Abstract

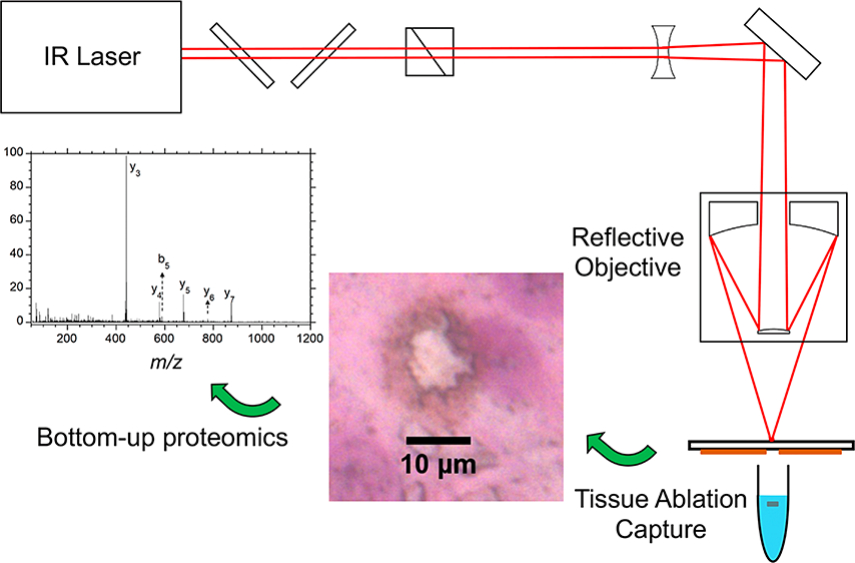

A Schwarzschild reflective objective with a numerical aperture of 0.3 and working distance of 10 cm was used for laser ablation sampling of tissue for off-line mass spectrometry. The objective focused the laser to a diameter of 5 μm and produced 10 μm ablation spots on thin ink films and tissue sections. Rat brain tissue sections 50 μm thick were ablated in transmission geometry, and the ablated material was captured in a microcentrifuge tube containing solvent. Proteins from ablated tissue sections were quantified with a Bradford assay, which indicated that approximately 300 ng of protein was captured from a 1 mm^2^ area of ablated tissue. Areas of tissue ranging from 0.01 to 1 mm^2^ were ablated and captured for bottom-up proteomics. Proteins were extracted from the captured tissue and digested for liquid chromatography tandem mass spectrometry (LC–MS/MS) analysis for peptide and protein identification.

## Introduction

Mass spectrometry is an important tool for probing biomolecular information in heterogeneous biological tissue. Localization, identification, and quantification of biomolecules in tissue sections by mass spectrometry can be achieved with imaging^1−4^ or by collection and off-line analysis of material from localized regions of interest (ROI).^5−10^ Mass spectrometry imaging excels at direct determination of the distribution of biomolecules within tissue sections with spatial resolution at micrometer scales. Mass spectrometry imaging (MSI) is rapid in part because it does not include a chromatographic separation step, but for this reason, quantification and identification are challenging. Efforts have been made to achieve confident identification by incorporating data-dependent acquisition into MSI^11−13^ and reliable quantification using various signal intensity normalization strategies.^14^ Fast gas-phase separation using ion mobility can be used to obtain some additional compound information^15−19^ but lacks the capabilities of liquid phase processing that are typically used in biological mass spectrometry. Furthermore, off-line analysis allows bottom-up protein sequencing by enzymatic digestion and liquid chromatography, which is one of the most powerful currently available methods for protein quantification and identification.^5,6,9,20,21^

Off-line mass spectrometry requires efficient removal and extraction of material and requires precise localization in the identified ROI for the most elucidating comparisons with mass spectrometry imaging. There are several methods for extraction from tissue for off-line LC–MS and LC–MS/MS analysis. Tissue sections can be manually dissected, for example, using a thin plastic film to assist in ROI selection for compound extraction.^22−24^ Commercial laser-based methods can be used to cut selected regions: laser microdissection uses a laser and an optical microscope system to select portions of a tissue section for removal.^25−27^ These systems use either an ultraviolet laser (UV) to cut the tissue or an infrared laser (IR) to melt a plastic film to physically capture the tissue but require extraction of biomolecules from the cells and tissue. Mass spectrometry can be performed directly from the microdissected regions^28,29^ or with liquid chromatography coupled with mass spectrometry.^21^ Because commercial microdissection methods collect largely intact tissue, extraction of the constituent biomolecules often involves tissue homogenization and cell lysis procedures.

Laser ablation is an alternative to laser microdissection that uses a focused pulsed laser to remove tissue through spot-by-spot laser irradiation of the ROI for collection and off-line analysis.^30−35^ Laser ablation is efficient at breaking up the tissue structure to facilitate extraction of the collected material, which obviates the requirement for cell lysis.^36^ In addition, laser ablation does not require coated slides or thermal polymer capture devices. The lasers for ablation and capture generally use UV or IR with wavelengths at which the sample can absorb the radiation. Mid-IR lasers with wavelengths ∼3 μm are efficient for ablation of biological tissue because of wavelength overlap with OH vibrational absorption of water molecules.^37^ Laser heating of the water in the tissue leads to a rapid volumetric phase change and ablation of the irradiated region, resulting in removal of the disrupted tissue; no significant biomolecule fragmentation has been reported.^38−42^ IR laser ablation and capture has been demonstrated for proteomic analysis from millimeter-sized tissue regions with a sampling spot size of approximately 200 μm.^43−45^

Laser ablation, with a small spot size that approaches the micrometer diffraction limit of a mid-IR laser, requires an objective with a large numerical aperture and a large working distance for viewing the tissue and collection of the ablated material. Single focusing elements are simple and provide a working distance of several centimeters but can only focus the laser to a spot size of approximately 100 μm.^34,38,46−48^ A typical multiple-element microscope objective has a high numerical aperture but small working distance that can be used in transmission mode ablation but otherwise limits access to the sample.^49−51^ A central hole can be cut through a high numerical aperture objective to allow the ablated material to pass through,^52−57^ but viewing the ablation process is otherwise obscured.

Reflective microscope objectives are widely used for infrared microscopy and have advantages of high numerical aperture, large working distance, and no chromatic aberration.^58−62^ Commercial reflective objectives are slightly larger than refractive objectives with diameters of several centimeters and similar working distances. A reflective objective with 8 mm working distance produced a 10 μm spot size with laser ablation electrospray,^63^ and an objective with 15 mm working distance achieved a 90 μm spot size with online liquid capture mass spectrometry.^32^ Reflective objectives have been constructed with larger diameters and working distances while maintaining high numerical aperture and small spot size. For example, a reflective objective with a 10 cm diameter concave mirror was used for 337 nm UV laser desorption with an FTICR mass spectrometer.^64^ The objective had a numerical aperture of 0.2 and working distance of 13 cm, which allowed direct ionization and UV postionization within the FTICR cell with an imaging resolution of 1 μm. A similar objective with 12 cm working distance was used for visible laser desorption and UV/visible postionization with a time-of-flight mass spectrometer and 1 μm resolution imaging.^65^

In the work described below, a large-format reflective objective was developed for mid-IR laser ablation and capture. The objective was constructed from gold-coated mirrors and has a 10 cm diameter and 10 cm working distance. The objective was used with a 3 μm optical parametric oscillator laser, and the spot size was measured by ablation of thin ink films and tissue sections. The objective was demonstrated with laser ablation sampling of rat brain tissue and proteomic characterization of the captured material. The ablated proteins were quantified with a Bradford assay and characterized with LC–MS/MS.

## Experimental Section

**Figure 1:**
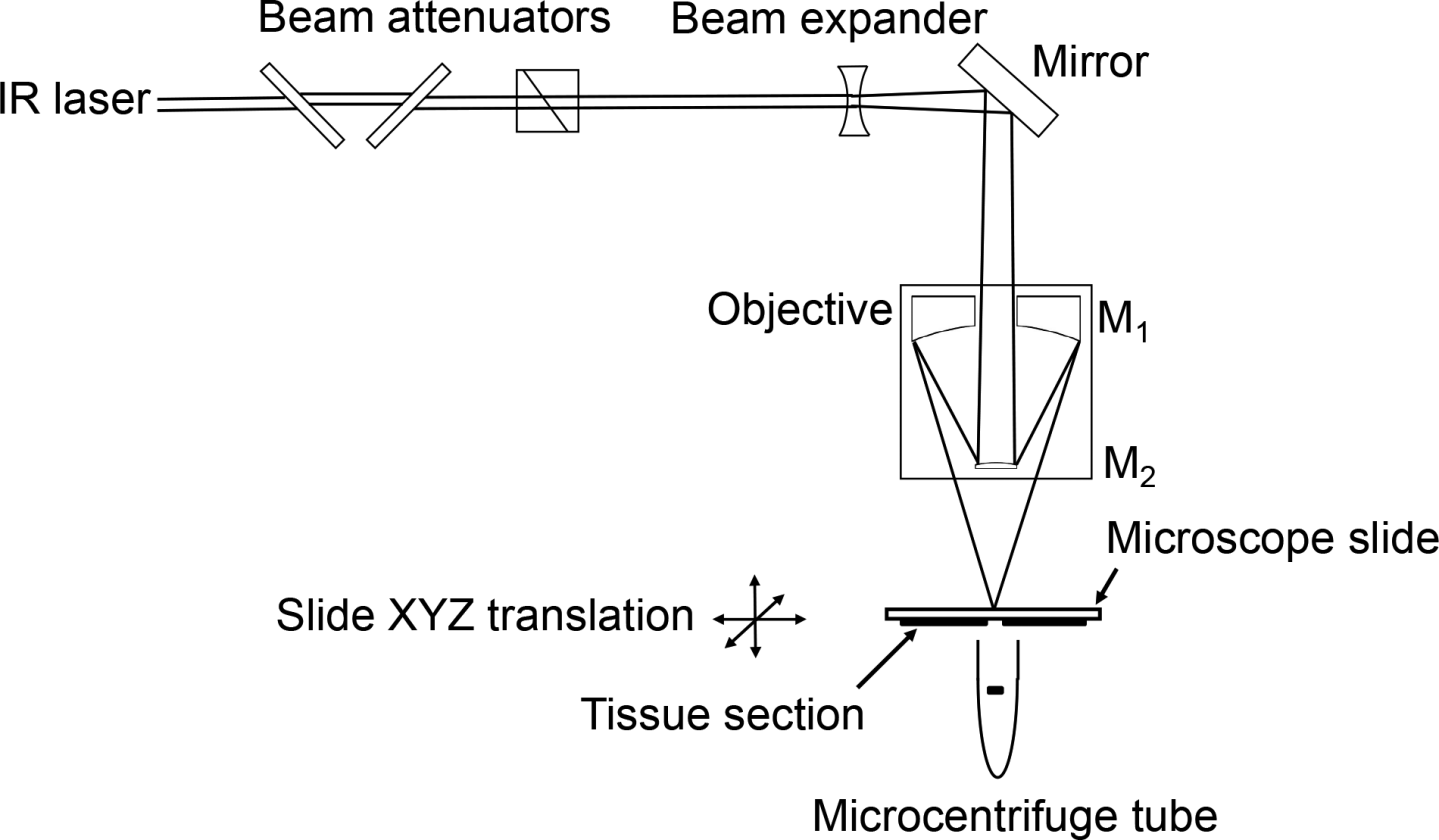
Schematic of the reflective Schwarzschild objective and laser sampling system.

The main components of the IR laser ablation sampling system have been described previously^44^ and comprise an OPO laser, attenuation and focusing optics, and a sample collection system. The configuration for the reflective objective system is shown schematically in Figure 1. The laser was a Nd:YAG pumped optical parametric oscillator (IR Opolette; OPOTEK, Carlsbad, CA) with a wavelength of 2940 nm, repetition rate of 20 Hz, pulse width of 7 ns, and maximum pulse energy of 3.5 mJ. The laser energy was attenuated using a ZnS dual rotating plate Brewster angle attenuator and a rotating CaF_2_ Glan-Taylor prism. The laser energy was adjusted between 2 and 300 μJ and measured with a thermocouple energy meter (M-Link, Gentec-Eo, Quebec City, Canada) before the slide.

The laser beam was expanded with a biconcave 25 mm focal length CaF_2_ lens and directed downward 90° to the objective using a 51 mm gold mirror. The Schwarzschild objective is based on a previously reported design^64,65^ and was constructed from a gold-coated 102 mm diameter concave mirror with a 25 mm diameter central hole (M_1_ in Figure 1) and a 25 mm diameter convex mirror (M_2_ in Figure 1). The radius of curvature was 135 mm for M_1_ and 52 mm for M_2_. The mirrors were mounted in a 130 mm length and 114 mm diameter cylindrical aluminum tube. The objective had a calculated numerical aperture of 0.3 and working distance of 98 mm.^60,66^ The large working distance facilitates sample positioning and visualization of the ablation process.

The focused laser diameter was measured using a single edge razor blade mounted on a 150 mm travel stage (ILS150PP, Newport, Irvine, CA) that was translated using a motion controller (XPS-Q8, Newport). The razor blade was translated across the focal plane in steps of 0.5 μm and the transmitted laser energy was measured using the energy meter and recorded as a function of razor blade position. The laser energy derivative was used to obtain full width at half-maximum (fwhm) of the beam profile using the GaussAmp fitting function in OriginPro (Version 2021, OriginLab Corporation, Northampton, MA).

Tissue sections were mounted on 1 mm thick soda-lime glass microscope slides with ∼65% transmission at 2940 nm and irradiated in transmission mode. The energy loss was used in the laser fluence reported below. The sample slide was translated on two horizontal axes for laser ablation and vertically to adjust the laser focus. The long axis was driven with a 150 mm travel stage (ILS150PP, Newport, Irvine, CA) and the short axis with a 50 mm stage (Model-433, Newport) and a motorized actuator (LTA-HS, Newport). The horizontal stages were operated by a motion controller (XPS-Q8, Newport) using custom LabVIEW (National Instruments, Austin, TX) software, and the vertical axis stage (Model-433, Newport) was manually operated.

Thin films of ink were created using a black ink indelible marker (Sharpie, Newall, Atlanta, GA) on a microscope slide. These films were translated using the long-travel stage and inspected using a microscope adjacent to the ablation area. The inspection microscope used a 10× objective (Model 43903, Edmund Optics, Barrington, NJ) with a USB camera (DCC1645C, Thorlabs, Newton, NJ). Optical images of samples were acquired with the USB camera or, after removal from the translation stage, with a stereo microscope (SteREO Lumar V12, Zeiss, Oberkochen, Germany) equipped with a 0.8× Neolumar S objective and a high-resolution digital camera (AxioCam HRx, Zeiss). Optical images obtained with the USB camera were processed using ImageJ (version 1.52a, National Institutes of Health, Bethesda, MD) to obtain ablation spot sizes. Image processing included converting images to 8-bit, thresholding the images into binary using the “Auto Threshold Default” to create white objects (ablation spots, above threshold) and black background (below threshold),^67^ and the “Analyze Particles” command to obtain the areas of object. Ablation spot diameters were calculated from the circle corresponding to the measured area.

Tissue sections were obtained from rat brain (Pel-Freez Biologicals, Rogers, AR) that was stored at −80 °C prior to sectioning. Coronal sections of fresh frozen rat brain were cut to 50 μm thickness at −25 °C using a cryostat (CM 1850, Leica Microsystem, Wetzlar, Germany) and thaw-mounted on glass microscope slides and stored at −80 °C until analysis. Thawed tissue sections were dried under vacuum for 3 min prior to IR laser ablation. Formalin-fixed and paraffin-embedded (FFPE) rat brain sections were prepared by immersing the whole tissue in 10% neutral buffered formalin (Sigma) for 48 h. The tissue was then cut in 4 mm thick slices along the coronal plane and mounted on paraffin embedding cassettes before immersion in paraffin (Sigma). The FFPE tissue was cut to 5 μm thick sections with a microtome (Leica RM2255), mounted on glass slides, and stained with hematoxylin and eosin (H&E, Anatech) using an automated tissue stainer (Leica XL).

Total protein quantification was performed with a Bradford colorimetric assay. Fresh frozen tissue sections were ablated in transmission mode and captured in a 300 μL microcentrifuge tube containing 200 μL of 10 mM ammonium bicarbonate buffer positioned ∼1 mm below the tissue slide, taking care to avoid laser irradiation of the surface of the capture solution. The 300 μL tube was aligned with the laser axis prior to ablation. For ablation of selected regions, the sample slide was translated in a raster pattern at 600 μm/s with 15 μm line spacing with the laser beam stationary. After ablation, a 150 μL volume of the buffer with captured tissue material was removed and added to a 96 well plate with 150 μL of the Bradford assay reagent solution (Coomassie Plus, ThermoFisher). The 300 μL mixture was incubated for 10 min at room temperature and the absorption measured with a microplate reader at 590 nm (Wallac 1420, PerkinElmer, Waltham, MA). A calibration curve was created using serial dilutions of a bovine serum albumin (BSA) standard for total protein quantification.

Proteins from the ablated and captured tissue were extracted and digested, and the resulting peptides were analyzed with LC–MS/MS for protein identification using a method described previously.^44^ A single-pot, solid-phase-enhanced sample-preparation (SP3) approach was used to digest the captured tissue.^44,68^ Ablated tissue was collected in a 300 μL microcentrifuge tube containing a 200 μL volume of 50 mM tris buffer (pH 8.5) with 1% sodium dodecyl sulfate (SDS, Sigma-Aldrich). Proteins in the ablated sample were reduced through the addition of dl-dithiothreitiol (DTT, Sigma-Aldrich) to each sample tube to a final concentration of 10 mM followed by incubation at 100 °C for 60 min. After cooling to room temperature, proteins were alkylated with iodoacetamide (IAA, Sigma-Aldrich) at a final concentration of 20 mM and incubated in the dark for 30 min. Paramagnetic beads were prepared by mixing two types of carboxylate modified magnetic particles (SpeedBeads and Sera-Mag, GE Life Sciences, Chicago, IL) at a ratio of 1:1 (v/v). The magnetic bead mixture was rinsed with and reconstituted in water at a concentration of 100 μg/μL. Two microliters of the bead solution was added to each sample tube after reduction and alkylation. Acetonitrile (ACN, VWR) was added to individual samples at a final concentration of 60% (v/v). The samples were incubated at room temperature for 20 min, and the beads in the sample were immobilized on a magnetic rack for 2–5 min until the supernatant remained clear. The beads were then rinsed on the magnetic rack twice with 70% ethanol and once with 100% ACN. The supernatant was discarded, and the beads were dried at 37 °C. The dried samples were resuspended and sonicated in 10 μL of 10 mM ammonium bicarbonate buffer.

Protein digestion was done by adding trypsin/lys-c mix (Promega, Madison, WI) to each tube at an enzyme to protein ratio of 1:25 (w/w) and followed by incubation overnight at 37 °C. After digestion, ACN was added to each sample to reach 95% (v/v) and incubated for 20 min. Sample cleaning was repeated twice with 70% ethanol and once with 100% ACN. The beads were incubated again on the magnetic rack for 2–5 min, and the supernatant was discarded. Peptides were eluted from the beads by adding 10 μL of 0.1% formic acid and sonicating for 5 min. The supernatant containing the peptides was collected after the beads were immobilized on the magnetic rack and then vacuum-dried.

Dried peptides from the protein digests were sent from Louisiana State University to Baylor University on dry ice and stored in a −80 °C freezer upon arrival prior to analysis. For MS analysis, the digests were brought to room temperature and reconstituted in 23 μL of LC injection solution, comprising 3% (v/v%) acetonitrile and 0.1% (v/v%) formic acid in water. Digested peptides were separated using a NanoACQUITY UPLC nanoflow reversed-phase liquid chromatography system (Waters, Milford, MA) in a single pump trapping configuration with a 20 μL partial loop injection, an ACQUITY UPLC M-Class Symmetry C18 trap column (100 Å pore size, 5 μm particle size, 180 μm × 20 mm), and an ACQUITY UPLC M-Class Peptide BEH C18 analytical column (130 Å pore size, 1.7 μm particle size, 100 μm × 100 mm). Mass spectrometry was performed with a Synapt G2-S HDMS (Waters) in positive-ion and resolution modes using traveling-wave ion mobility spectrometry (TWIMS)-enhanced MS^E^ and drift time-dependent collision-induced dissociation (CID) energy ramps (UDMS^E^).^69^ The UPLC-UDMS^E^ method followed previous studies^20,44^ with peptides trapped at 7 μL/min flow for 6 min and separated at 300 nL/min for 90 min.

UDMS^E^ data were processed with ProteinLynx Global Server (PLGS V. 2.5.2, Waters) using modified “Electrospray MS^E^” parameters: “Low Energy” and “Elevated Energy” ion detection thresholds were set to 100 and 50 counts, respectively, to facilitate low-signal detection. Peptide precursor and fragment ion signals were correlated during processing by aligning ion mobility arrival times with LC retention times and the peptides were identified using the PLGS Ion Accounting algorithm. Sequences generated from correlated fragment ions were matched against the UniprotKB/Swiss-Prot and UniprotKB/TrEMBL *Rattus norvegicus* protein database (Uniprot 2020_04) containing 31577 protein entries. Default PLGS ion accounting parameters were used, apart from a wider 0.5 Da lock mass window to compensate for mass drift during prolonged acquisitions (>24 h). Peptide and fragment mass tolerances were optimized automatically and 95% of peptide precursor ions had mass measurement error less than 6 ppm. Putative trypsin digest sites were used for determination of theoretical digest peptide sequences. Carbamidomethyl-modified cysteine was set as a fixed modification, and oxidized methionine as a variable modification. The maximum number of missed cleavages was set to 2. Peptides identified by PLGS with more than 5 amino acids and nonzero score were used for BLAST analysis with in-house software (Protein and Imaging Tools (PIT), ver. 1.0.4) and searched against the UniprotKB/TrEMBL and UniprotKB/Swiss-Prot *Rattus norvegicus* protein database. Proteins with two or more matching (but not necessarily unique) peptides were considered identified.

## Results and Discussion

Initial experiments were performed to measure the diameter of the focused IR laser beam and the size of laser ablation spots. The focused beam was measured using a translated single-edge razor blade at the laser focal plane that was 10 cm from the convex mirror of the Schwarzschild objective. The plots of transmitted laser energy and its derivative as a function of razor blade position are shown in the Supporting Information (Figure S1), and the fit of the derivative plot representing the Gaussian beam profile at the laser focal point indicates a fwhm of 5 μm.

**Figure 2:**
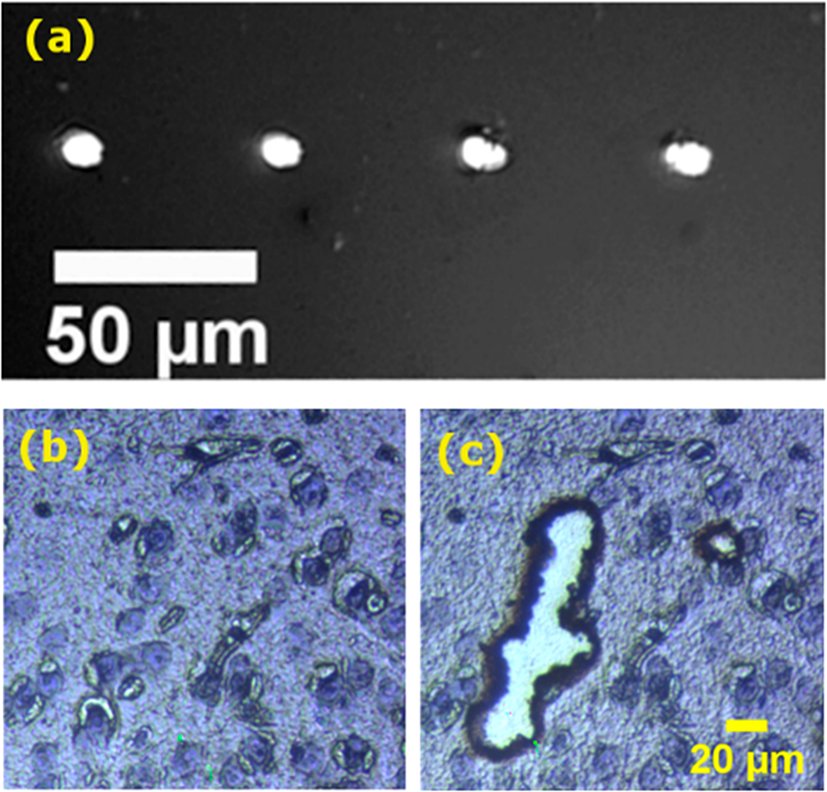
Optical microscope images of (a) four laser-ablated spots on a thin film of ink at 6 μJ and (b) 5 μm thick H&E stained rat brain tissue before and (c) after ablation at 6 μJ with approximately 100 laser pulses.

The diameter of laser-ablated spots created with a single laser shot in thin ink films on a microscope slide was measured at laser energies of 6, 8, 10, 20, 60, 100, 140, and 180 μJ. Coarse laser energy attenuation was achieved with the rotating plate attenuator, and fine laser energy attenuation was achieved using the rotating prism. The ablation spot diameters were measured using optical microscope images and ImageJ software. At 4 μJ and below, no indication of ablation was observed. The spot at 6 μJ energy had a diameter of 9.3 μm ± 0.4 μm (*n* = 4), shown in Figure 2a, corresponding to a fluence of 88 kJ/m^2^. The ablated spot radius increased logarithmically with laser energy (Figure S2), consistent with a Gaussian laser profile. The 2940 nm ablation spot diameter of 9.3 μm at the ablation threshold was approximately 10 times larger than that previously obtained with a similar Schwarzschild objective and a 337 nm UV laser,^64^ which is consistent with the 10 times longer IR wavelength.

Single-spot and region ablation were demonstrated with 5 μm thick H&E stained FFPE tissue sections mounted on a microscope slide. Stained tissue sections were used to aid in locating cells for ablation. The tissue section position was adjusted to the laser focus prior to ablation. No ablation was observed at 4 μJ laser energy or below, and analogous to the ink film, the ablated spot diameter was approximately 10 μm at 6 μJ and 50 μm at 180 μJ. Ablation of a region on stained FFPE tissue is shown in Figure 2: Figure 2b shows a region of tissue before ablation and Figure 2c shows the region after ablation of several selected cells at an energy of 6 μJ.

Fresh frozen tissue sections were vacuum-dried and ablated, and the captured proteins were quantified with a Bradford colorimetric assay. Rat brain tissue sections 50 μm thick were ablated at 6, 13, 26, 50, 80, 100, 130, 160, and 180 μJ with three replicates at each laser energy. The ablated spot size in tissue was comparable to that observed for ablation of the ink films (Figure S3), and the minimum fluence for ablation was 71 kJ/m^2^ with 10.4 ± 0.9 μm spot diameter (*n* = 4). The total captured protein from 1 mm^2^ regions was quantified, and the results are shown in Figure S4. No protein was detected in the captured material ablated at 6 and 13 μJ laser energies; however, a 125 ng quantity of protein was obtained with 26 μJ, and 300 ng protein was obtained with 50 μJ. The regions ablated at 6 and 13 μJ appeared to have residual tissue material that was not completely removed by the laser. There was a slight decrease in the amount of captured protein at energies above 50 μJ, which could result from heating and protein degradation at higher pulse energies or from less efficient capture under more vigorous ablation conditions. The 300 ng protein corresponds to approximately 50% capture efficiency and is comparable to that reported previously for IR laser ablation and transfer.^36^ This is comparable to the capture efficiency of laser microdissection which can vary from 40 to 90%.^70,71^

The fluence required for ablation using the reflective objective and 10 μm spot size is approximately five times that reported using a single focusing element with a 250 μm spot size.^20,36,41,42^ Relatively lower ablation efficiency for smaller spot sizes has been observed previously for infrared laser ablation of tissue and has been attributed to the additional energy necessary to overcome the tissue tensile strength with a low aspect ratio ablated area.^72,73^

**Figure 3:**
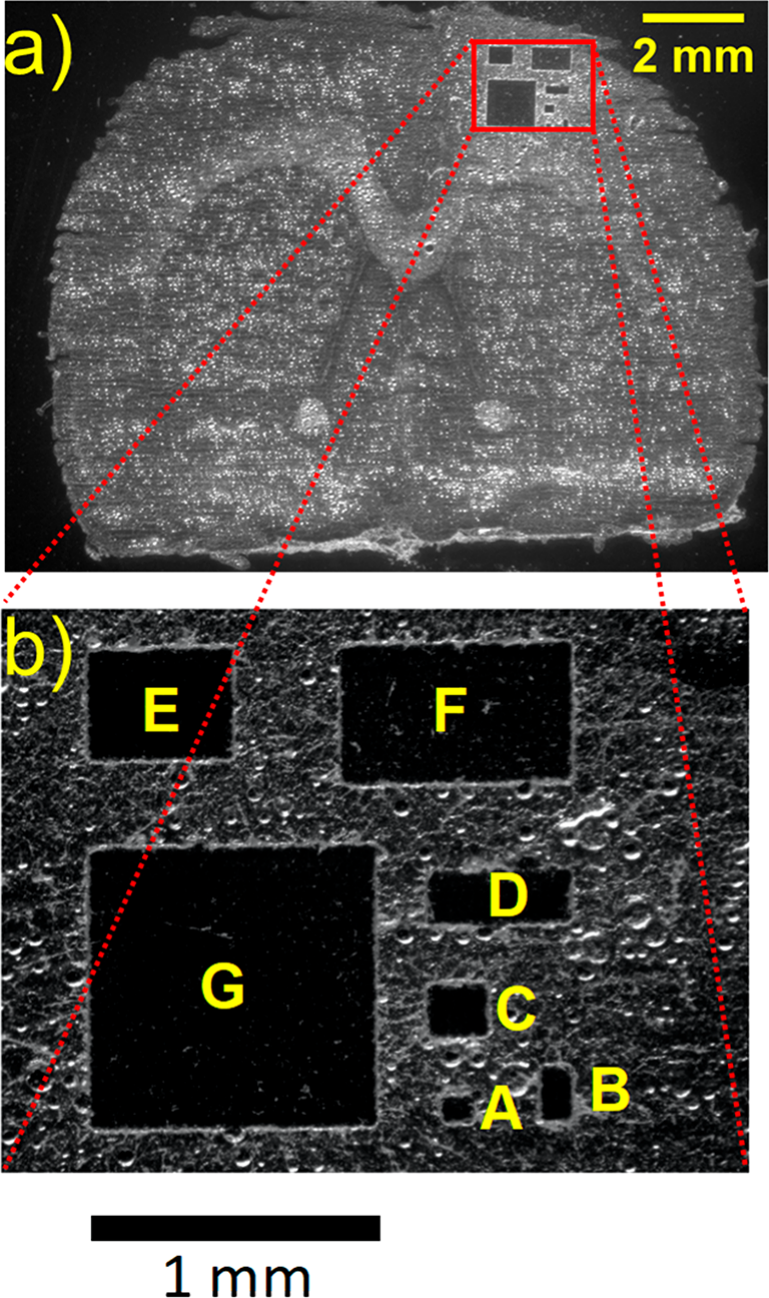
(a) Optical microscope image of a rat brain tissue section after IR laser ablation; (b) expanded region showing ablated areas A (0.01 mm^2^), B (0.02 mm^2^), C (0.04 mm^2^), D (0.1 mm^2^), E (0.2 mm^2^), F (0.4 mm^2^), and G (1 mm^2^).

Seven areas with a range of sizes were ablated from fresh frozen rat brain tissue sections at a laser energy of 50 μJ, and proteins in the captured material were digested for analysis by LC–MS/MS. Three replicates for each area were obtained from three consecutive 50 μm thick tissue sections; the seven areas in each tissue section were located within a 2 × 2 mm^2^ region of the cerebral cortex region of a single rat brain (Figure 3a). Figure S5 shows the orientation of the areas on each tissue section. The regions were randomly distributed in the first tissue section (Figure 3), and then their location in the second and third tissue sections (Figure S6a,b) was determined by serial 90° counterclockwise rotations. The ablated areas were 0.01, 0.02, 0.04, 0.1, 0.2, 0.4, and 1 mm^2^ and are indicated as A–G, respectively, in the optical image in Figure 3b and Figure S6. Ablated tissue was collected in tris buffer, digested with trypsin overnight to generate peptides, and then dried under vacuum and stored at −80 °C prior to LC–MS/MS analysis. Dried tryptic peptides were reconstituted in 23 μL of LC injection solutions and 20 μL was loaded onto a trap column and separated with a nanoLC C18 analytical column using a gradient flow of 300 nL/min over 90 min.

**Figure 4:**
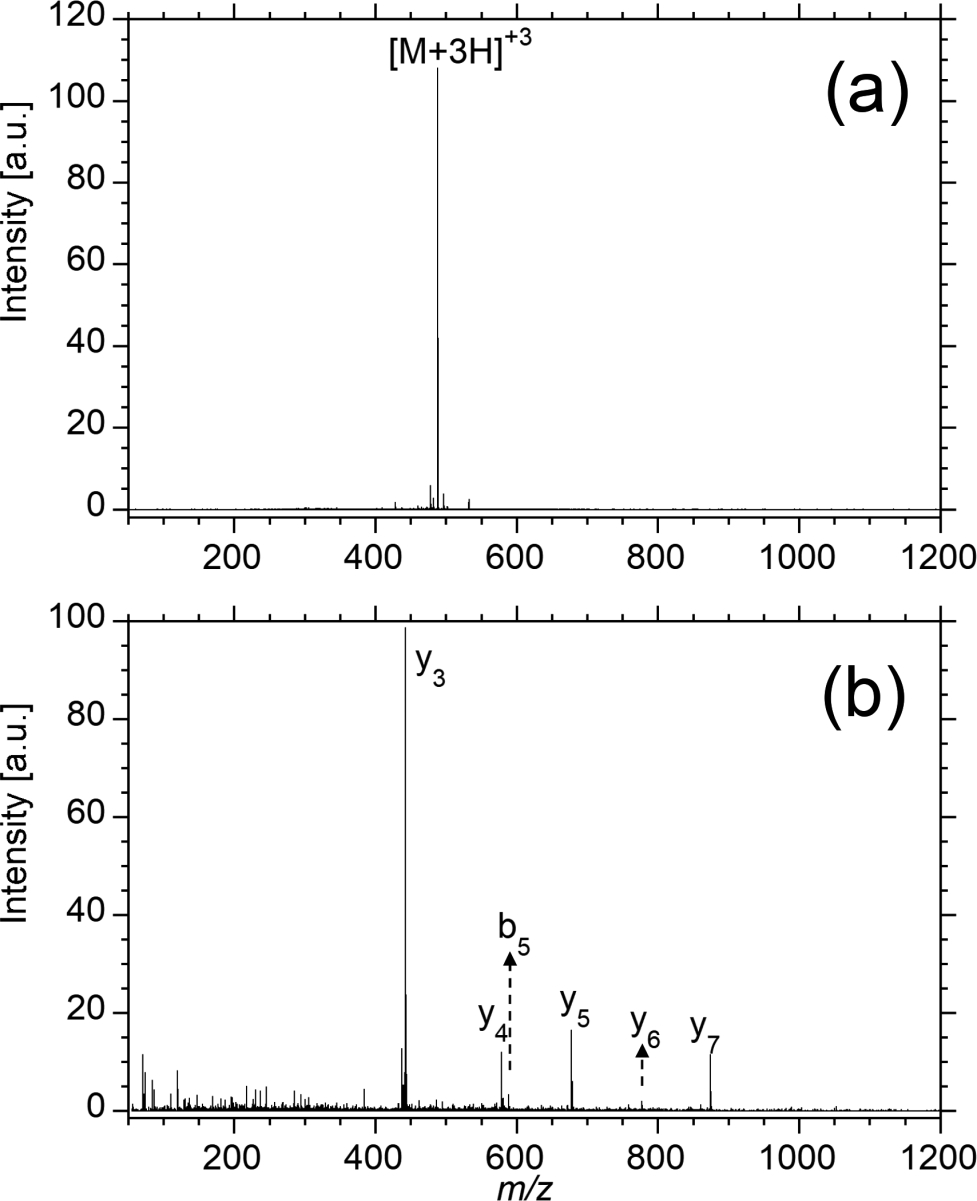
(a) Representative mass spectrum and (b) MS/MS spectrum from UPLC–HDMS^E^ data for the trypsin fragment of myelin basic protein (unique peptide TQDENPVVHFFK) from tissue analysis of smallest ablated area C (0.04 mm^2^).

The LC eluate was analyzed by UDMS^E^ mass spectrometry using a Synapt G2-S mass spectrometer. The UDMS^E^ data was processed with PLGS for peptide identification, and those containing at least five amino acids were searched against the UniprotKB/TrEMBL and UniprotKB/Swiss-Prot *Rattus norvegicus* protein database to identify matching proteins: those with at least two matching peptides were considered identified. A representative mass spectrum of a myelin basic protein tryptic peptide from the 0.04 mm^2^ region is shown in Figure 4a, and the MS/MS spectrum from the triply charged precursor is shown in Figure 4b. The peptide was identified as TQDENPVVHFFK, which is common to all isoforms of myelin basic protein.

The average number of proteins and peptides identified in each ablated region is shown in Table 1. An average of 6, 70, 240, 530, and 890 proteins was identified in the 0.04, 0.1, 0.2, 0.4, and 1 mm^2^ areas, respectively. The coefficient of variation in the number of detected proteins and peptides among the ablation area triplicates was approximately 16%. The molecular weight of the detected proteins in all areas ranged from 6 to 550 kDa. Over 80% of the total identified proteins in all areas had molecular weights less than 100 kDa and less than 2% proteins had molecular weight greater than 300 kDa (Figure S7a). The protein abundance had a range of approximately 2 orders of magnitude. The ranges of both protein molecular weight and protein abundance decreased with decreasing ablation area (Figure S7), which is expected due to the lower protein content from the smaller areas. Although photofragmentation of the ablated proteins is not anticipated, the IR laser ablation could potentially reduce the number of identified proteins by reduction of tryptic peptides and production of peptides with unanticipated backbone or side chain cleavages.

**Table 1:** Proteins and Peptides Identified in Tissue Regions

region label	area (mm^2^)	proteins	peptides
A	0.01	0	0
B	0.02	0	0
C	0.04	6 ± 2	20 ± 4
D	0.1	70 ± 6	110 ± 15
E	0.2	240 ± 50	400 ± 80
F	0.4	530 ± 40	1100 ± 80
G	1	890 ± 110	2100 ± 400

The number of proteins identified in the 1 mm^2^ ablated area of 50 μm thick brain tissue was compared to that obtained in previous studies: 890 in this work and approximately 400 in previous studies.^20,36^ The larger number of proteins identified in this work is likely due to the more efficient SP3 method compared to the filter aided sample preparation (FASP) method that was used in the previous studies. For the smaller ablated areas, the sensitivity limitations of the SP3 approach, as currently implemented, are apparent. Only six proteins on average were identified in the 0.04 mm^2^ ablated tissue area, which contains approximately 12 ng of protein. Low volume sample processing techniques are currently able to process sub-nanogram quantities of protein.^74−76^ Adaptation of such low volume methods to small area laser ablation will be necessary for successful proteomic analysis at the size capability of the reflective objective.

## Conclusions

A reflective objective was developed for IR laser ablation that has a 10 cm working distance and 5 μm focused beam fwhm and is capable of ablating 10 μm diameter spots in tissue sections. A transfer efficiency of 50% was achieved as assessed with a Bradford assay of the captured material. Regions from 50 μm thick rat brain tissue sections were ablated and captured for bottom-up mass spectrometry analysis. Proteins were identified in areas down to 0.04 mm^2^ containing approximately 12 ng protein, which represents the practical lower bound for the protein analysis method employed. Future work is aimed at adapting low volume sample processing to proteomics of small area ablation and capture of tissue.^77−79^

## Supporting Information Available

Focused beam diameter measurement using knife edge scan method (Figure S1), laser ablation spot radius on ink films as a function of laser energy (Figure S2), laser ablation spot radius on 50 μm thick tissue as a function of laser energy (Figure S3), Bradford assay protein content of ablated rat brain tissue as a function of laser energy (Figure S4), laser ablation area orientation in rat brain tissue (Figure S5), optical microscopic images of two consecutive rat brain tissue sections (Figure S6), and molecular weight and abundance distributions of proteins in the ablated tissue (Figure S7) (PDF)
