# Supplementary material for: Infrared Laser Ablation Microsampling with a Reflective Objective
Source: J Am Soc Mass Spectrom. 2022 Feb 1;33(3):463–70. doi: 10.1021/jasms.1c00306 (PMC8895455; doi:10.1021/jasms.1c00306)
Supplement: Supplementary file 1 — js1c00306_si_001.pdf [file js1c00306_si_001.pdf]

Infrared Laser Ablation Microsampling with a Reflective Objective

Chao Dong[1]; Luke T. Richardson[2]; Touradj Solouki[2], Kermit, K. Murray[1]*

[1]Department of Chemistry, Louisiana State University, Baton Rouge, Louisiana. 70803,
[2]Department of Chemistry and Biochemistry, Baylor University, Waco, Texas, 76706
United States

Supporting Information

*Corresponding Author and reprint requests: Dr. Kermit K. Murray,
331 Choppin Hall,
Department of Chemistry,
Louisiana State University,
Louisiana, 70803, United States
Phone: +1 (225) 578-3417
Fax: +1 (225) 578 3458
E-mail:kkmurray@lsu.edu

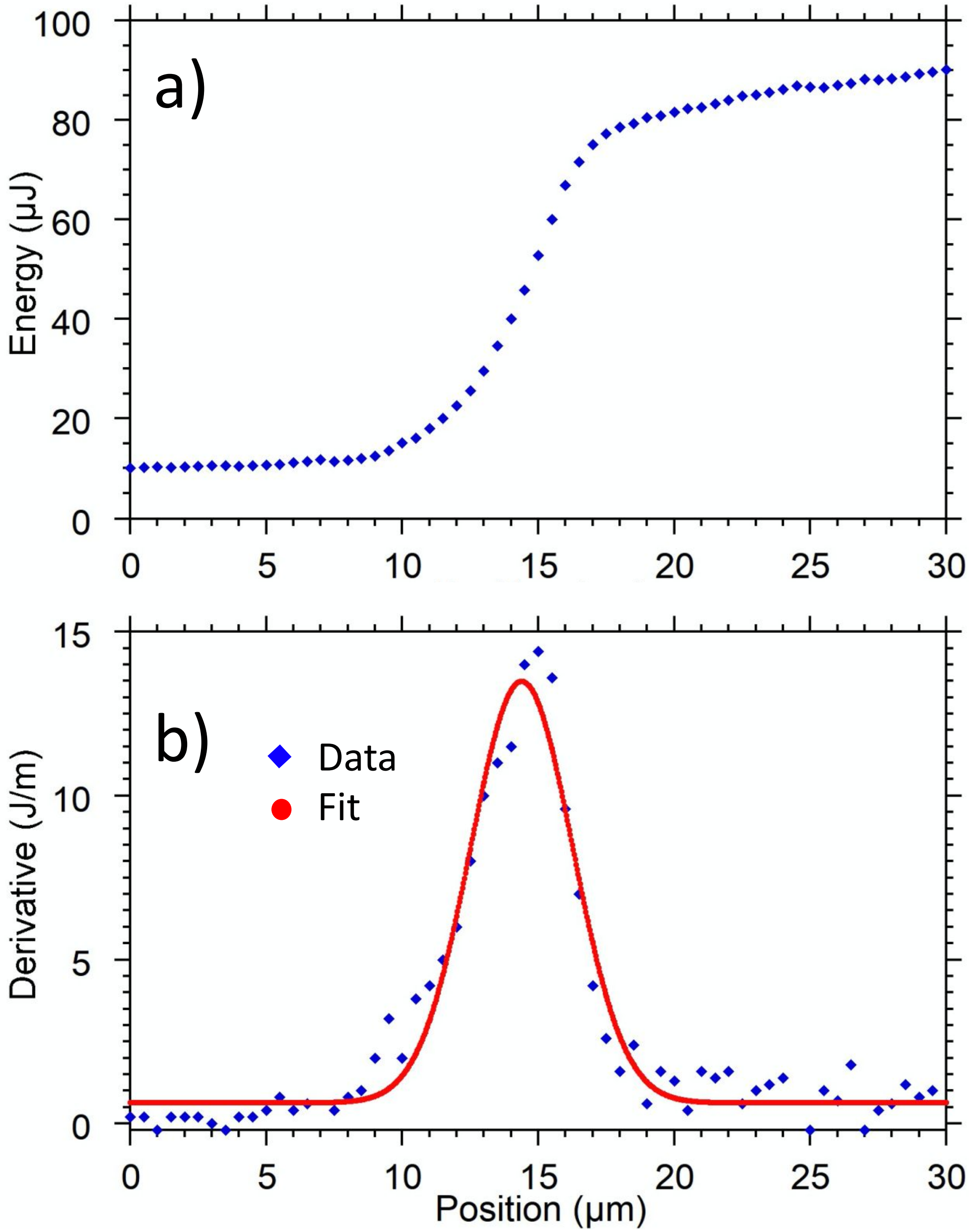

Figure S1. (a) Transmitted laser energy from a knife edge scan across at the laser focal plane and (b) derivative with FWHM of 5 μm representing the beam profile for the fit.

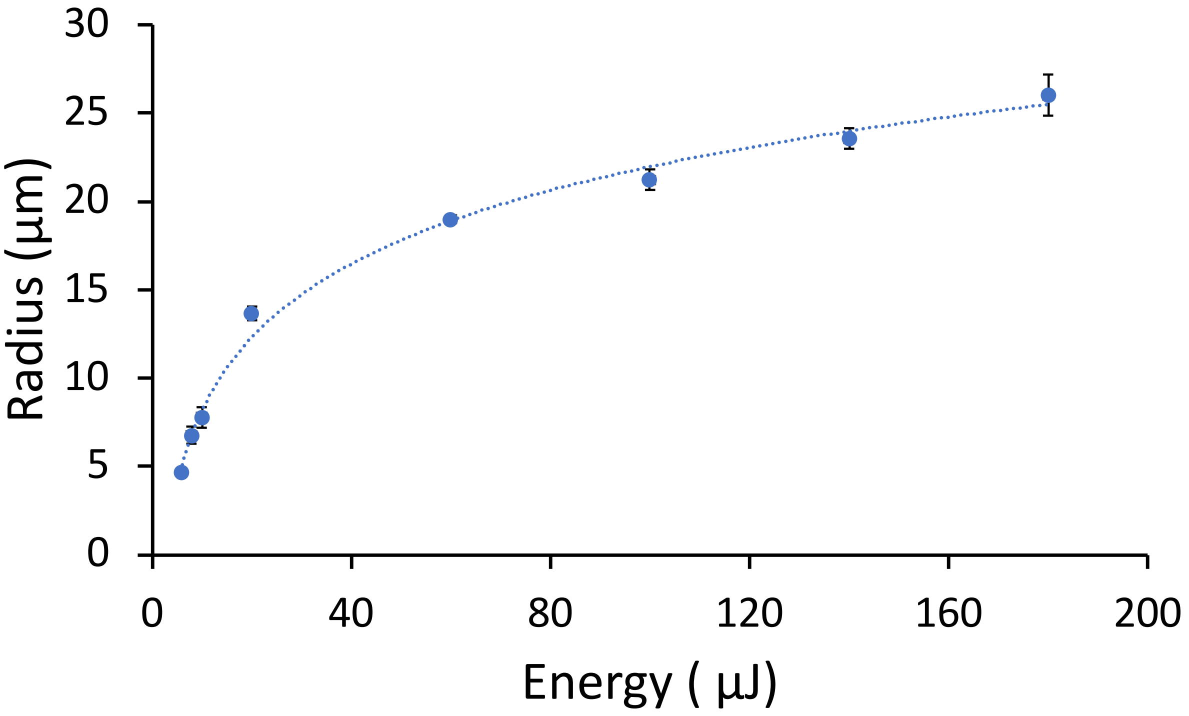

Figure S2. Ablation spot radius on ink as a function of laser energy. The radius and energy fits to the equation y = a*ln (x) + b. Error bars indicate one standard deviation (n=4).

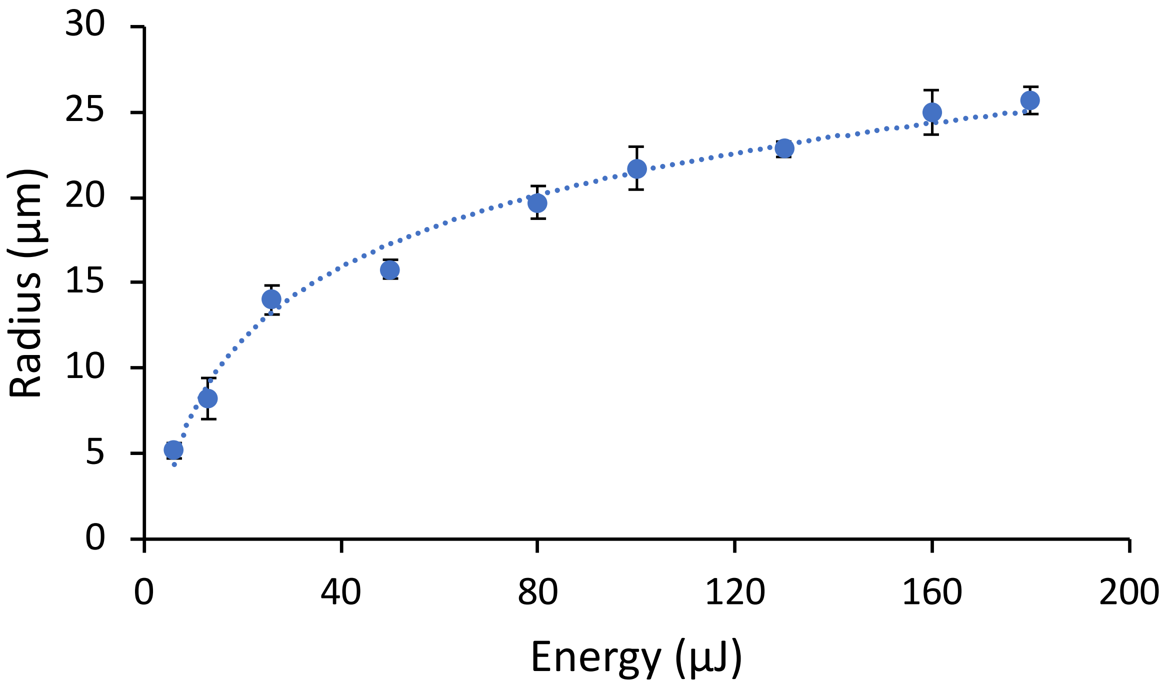

Figure S3. Ablation spot radius on 50 µm thick tissue as a function of laser energy. The radius and energy fits to the equation y = a*ln (x) + b. Error bars indicate one standard deviation (n=4).

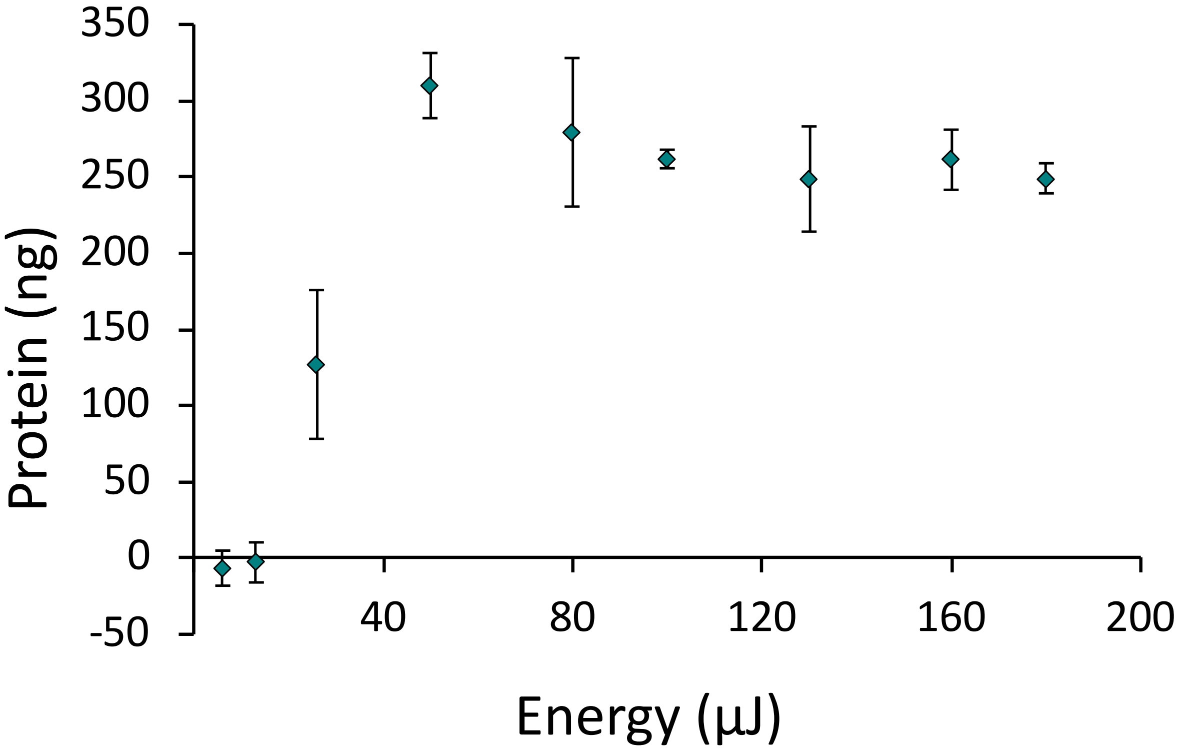

Figure S4. Bradford assay protein content from a 1 mm^2 area of ablated rat brain tissue as a function of laser energy. Error bars indicate one standard deviations (n=3). No protein was detected at 6 and 8 μJ.

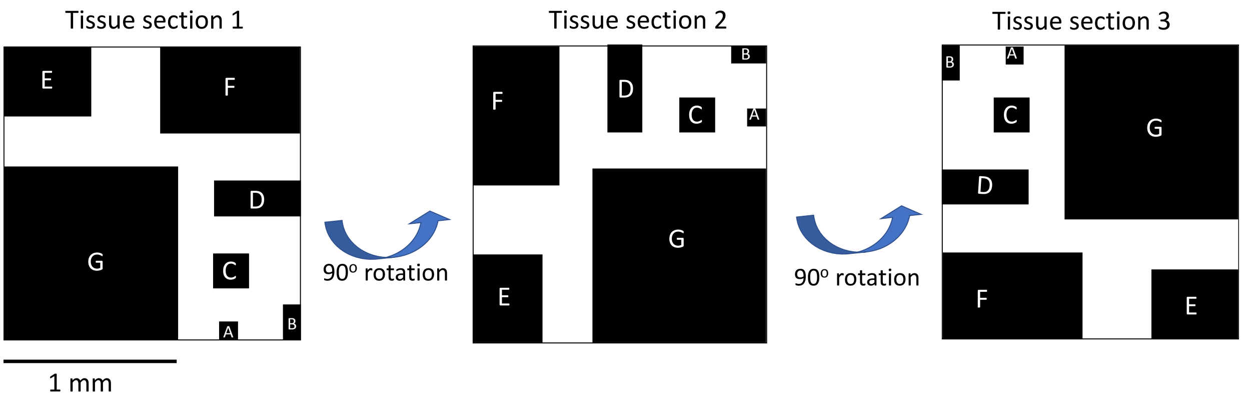

Figure S5. Schematic indicating the location of ablated areas A-G in three consecutive tissue sections. The areas were distributed in a 2 mm x 2 mm region and their orientation determined by successive 90º counterclockwise rotation about the center of the pattern.

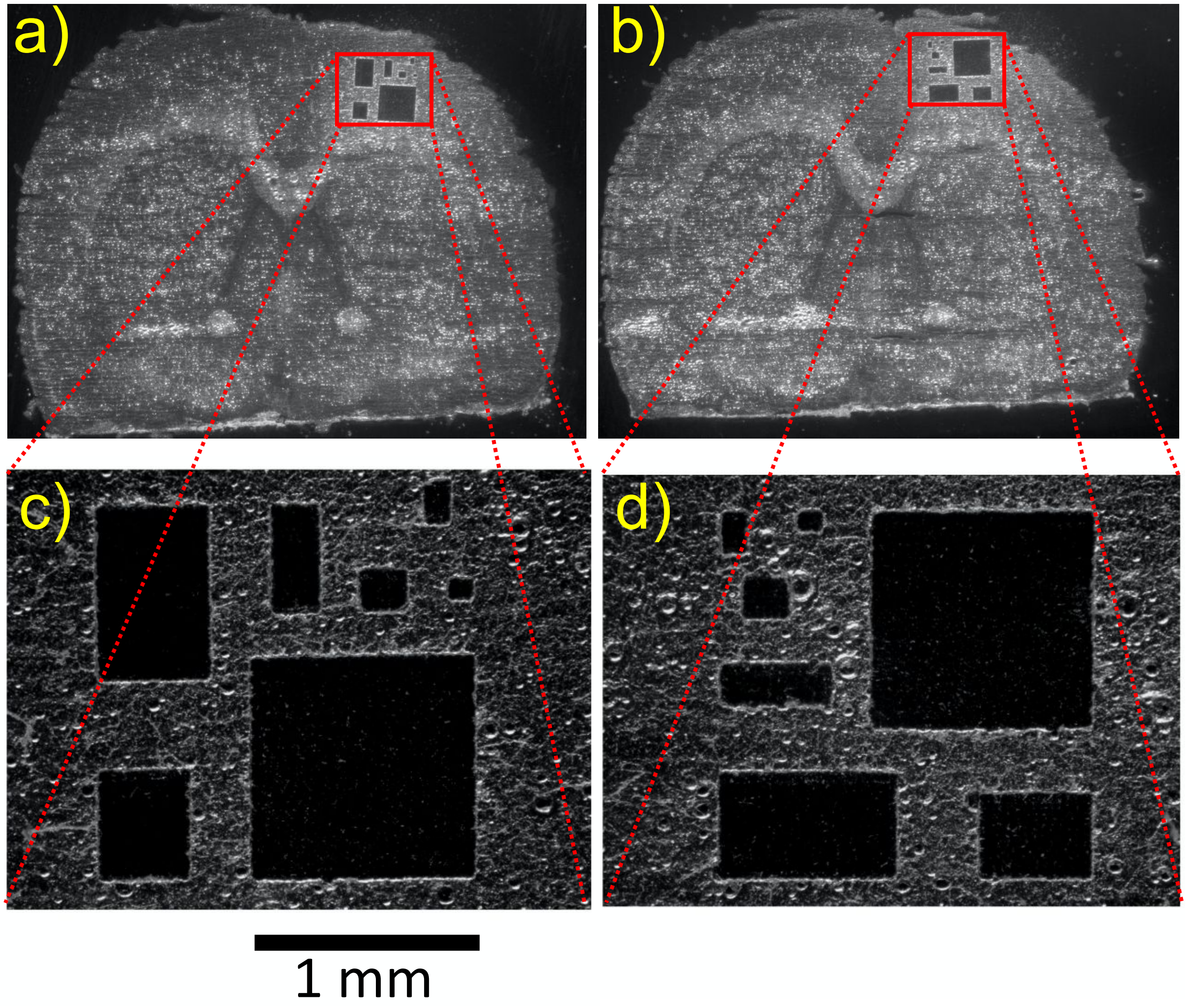

Figure S6. Optical microscopic images of two consecutive 50 μm rat brain tissue sections (with Fig 3b) from the cerebral cortex region after laser ablation (a and b), and the zoomed-in regions (c and d).

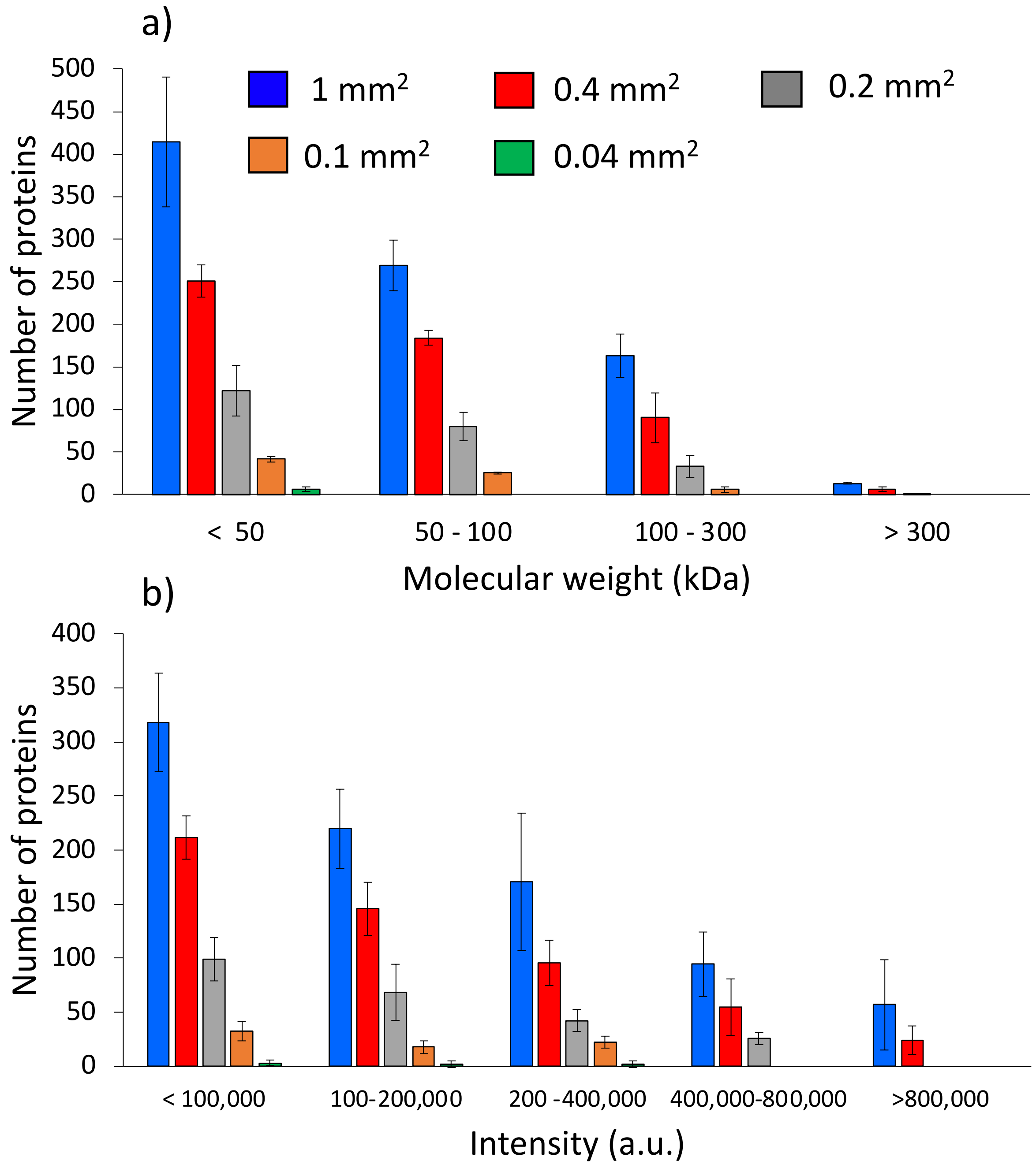

Figure S7. Molecular weight and abundance of proteins identified in the ablated rat brain tissue areas: 1 mm^2, 0.4 mm^2, 0.2 mm^2, 0.1 mm^2 and 0.04 mm^2 with a) number of proteins in molecular weight ranges <50 kDa, 50-100 kDa, 100-300 kDa, and > 300 kDa and b) number of proteins in peak intensity ranges < 100, 000, 100,000-200,000, 200,000-400,000, 400,000-800,000 and > 800,000 arbitrary units. Over 80 % of the total identified proteins in all areas had molecular weight less than 100 kDa and less than 2% proteins had molecular weight greater than 300 kDa. The ranges of both molecular weight and protein abundance decreased with decreasing ablation area. The error bars indicate one standard deviation (n=3).
